# Rutin Improves Anxiety and Reserpine-Induced Depression in Rats

**DOI:** 10.3390/molecules27217313

**Published:** 2022-10-27

**Authors:** Ahmed I Foudah, Mohammed H Alqarni, Aftab Alam, Sushma Devi, Mohammad A Salkini, Prawez Alam

**Affiliations:** 1 Department of Pharmacognosy, College of Pharmacy, Prince Sattam Bin Abdulaziz University, P.O. Box 173, Al-Kharj 11942, Saudi Arabia; 2 Chitkara College of Pharmacy, Chitkara University, Rajpura 140401, Punjab, India

**Keywords:** rutin, antidepressant, depression, anxiety, reserpine, mental disorders

## Abstract

Mental disorders have a poor clinical prognosis and account for approximately 8% of the global burden of disease. Some examples of mental disorders are anxiety and depression. Conventional antidepressants have limited efficacy in patients because their pharmacological effects wear off, and side effects increase with prolonged use. It is claimed that herbal medicine’s antioxidant capacity helps regulate people’s mood and provide a more substantial pharmacological effect. With this background, the purpose of this study is to investigate the effect of rutin on reserpine-induced anxiety and depression in rats. The animals were divided into groups of six rats each: normal control (water), a depression model, a rutin-treated rat model, and an amitriptyline-treated rat model. According to the results, 14 days of treatment with rutin, once daily, showed a modest antidepressant effect. This effect was mediated by increased serotonin, norepinephrine, and dopamine levels in cortical and hippocampal regions. The antioxidant and vasodilator properties of rutin may contribute to its antidepressant properties. According to this study, rutin has shown antidepressant effects by reducing antioxidant activity and acetylcholinesterase.

## 1. Introduction

Mental diseases known as major depressive disorders (MDDs) are characterized by a general disinterest in and inability to enjoy pleasurable activities. MDD has a worldwide incidence of 4.7% and is one of the most severe mood disorders, associated with a high suicide rate and significant direct and indirect functional impairment [1]. Major depression is a common disorder that significantly limits psychosocial functioning and reduces quality of life. According to the World Health Organization (WHO), major depression ranked as the third greatest cause of clinical suffering throughout the globe in the year 2008; however, the WHO projects that this condition would top the list of causes of clinical distress by the year 2030 [2]. Depression is more likely to occur in women than to men over the course of a woman’s lifetime. Depression has also been linked to an increased risk of cardiovascular disease, cerebral cardiovascular disease, and other forms of mortality due to diseases connected with the heart and blood vessels [3].

The etiology of depression remains complicated; however, studies suggest that the serotonergic, dopaminergic, and noradrenergic pathways play a role [4]. Depression is a complex disease, and the differences in the diagnosis of depression are even similar in two people [5]. Environmental, congenital, and genetic variables play an important role in the development of MDD symptoms. Diagnosis, treatment, and prevention of depression remain important public health goals. Traditionally, pharmacotherapy and psychotherapy are used to treat depression and anxiety disorders. Antidepressants and anxiolytics modulate neurotransmitters in the central and peripheral nervous systems [6]. Most synthetic chemical drugs are currently used to treat depression. Benzodiazepines are one of the most common and effective drugs used around the world to treat anxiety. In addition, barbiturates and benzodiazepines are commonly used. The effects of barbiturates and benzodiazepines on the central nervous system are mediated by interactions with the GABA receptor [7]. However, early antidepressant treatment may not provide adequate relief for the patient, due to the drug’s adverse effects of the drug or its lack of instant efficiency. According to current standards, treatment of major depression involves relief and restoration of functioning. Therefore, new agents that have an improved safety profile in addition to increased efficacy [8]. Natural therapies offer advantages over synthetic chemical drugs in the prevention and treatment of depression. Herbal treatments may provide an alternative to synthetic antidepressants that offers a more favorable balance between potential benefits and possible adverse effects [9].

Herbal remedies are called green medicine because they are harmless and effective. Traditional herbal treatments have been shown to be effective in treating depression. Traditional herbal treatments are widely known to be a viable source of possible active components for the development of more effective and less toxic therapeutics [10]. Due to their remarkable pharmacological benefits, cost effectiveness, and lack of side effects, traditional herbal medicines have gained prominence in numerous healthcare settings in recent decades [11].

Rutin is a flavonoid diglycoside also known as sophorin or quercetin-3-rutinoside. It contains the deaccharide rutinose and the flavonoid quercetin. It is a powerful antioxidant and anti-inflammatory flavonoid found in a variety of fruits and vegetables such as walnut kernels, flowers, onions, oranges, apples, lemons, grapes, tea and red wine. Other sources include walnut kernels, flowers and onions [12]. It has been shown to exert several biological activities, such as antimicrobial, anti-inflammatory, antioxidant, neuroprotective, antiviral, and antiulcerogenic [13,14]. Rutin has been shown to be a potential neuroprotector in vivo and in vitro [15,16,17,18].

Rutin may have beneficial effects on anxiety-related depression and changes in learning and memory behavior, but this has not yet been explored. Therefore, the aim of our study was to evaluate the efficacy of rutin as an antidepressant in rats administered reserpine to induce depression. Considering the properties of rutin and the fact that the specific mechanisms behind its beneficial effects are not yet known, the study is very comprehensive. Given its comprehensive nature, the study of rutin and its antidepressant effects is essential for contemporary treatment. In view of the information presented above, the aim of this study was to investigate the effect of rutin on reserpine-induced depression and anxiety in rats.

## 2. Results

### 2.1. Effect of Rutin Pretreatment on Body Weight

**Figure 1:**
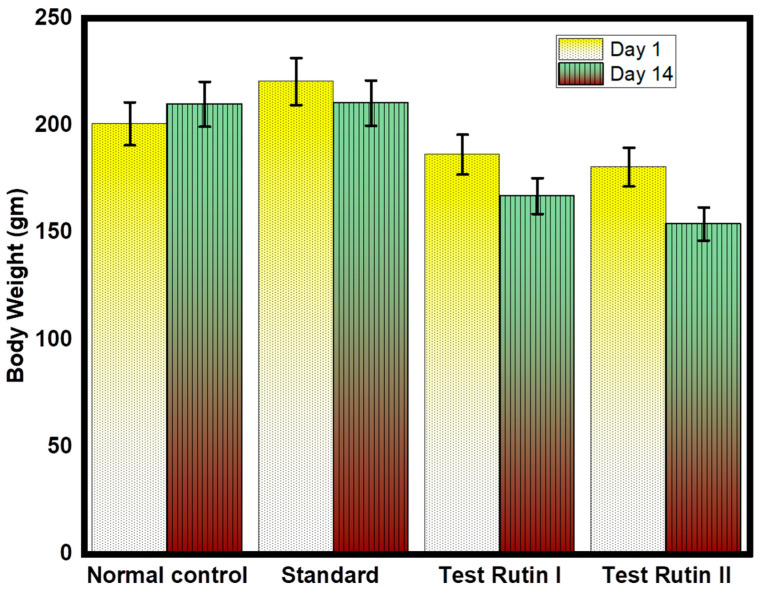
Effect of rutin pretreatment is compared to the initial and final weight of the rat. The results are expressed as mean ± SD values (*n* = 6). The significance of the difference was determined by one-way ANOVA; * *p* < 0.05 vs. respective controls.

Rutin is a phenolic compound found in several plants. The study investigated the effects of rutin on obesity in depressed rats. To study the effect of rutin on body weight, five groups of rats were fed with or without samples for 7 weeks. Figure 1 shows changes in body weight. At the beginning of the experiment, there was no significant difference in the initial weight of the group. All three groups (normal control, standard and negative control) had a substantial increase in body weight on or near the last day of the trial (*p*  <  0.01). In the group treated with different rutin doses, weight gain decreased significantly. Researchers have reported on the anti-obesity effects of rutin, including weight loss, and various researchers have reported on the effect of rutin on weight loss [19,20]. According to the results, the effect of different doses of rutin 80 mg/kg on the reduction of body muscle was greater than that of rutin 40 mg/kg. The impact of normal and standard weight reduction is statistically not significant. However, the effect of 80 mg/kg rutin seems to be greater than the administration of 40 mg/kg rutin alone. Therefore, the other possible effect of rutin’s anti-obesity can be linked to antioxidant activity. However, more research is needed to clarify the exact mechanisms.

### 2.2. Effect of Rutin Pretreatment on Antianxiety Activity

#### 2.2.1. Elevated Plus Maze (EPM)

**Figure 2:**
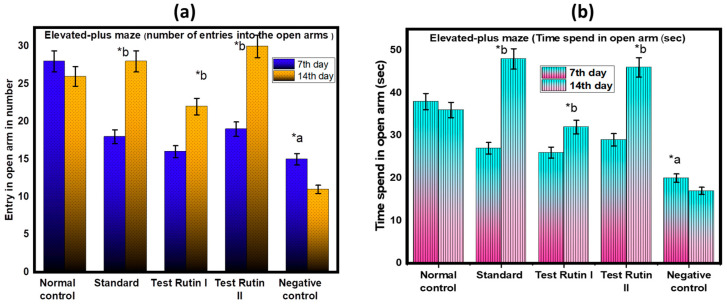
Effect of rutin pretreatment on antianxiety activity in rats using EPM: (**a**) EPM (number of entries into the open arms); (**b**) Time spend in open arm (sec). The results are expressed as mean ± SD values (*n* = 6). The significance of the difference was determined by two-way ANOVA; * *p* < 0.05 vs. respective controls. a = normal control vs. reserpine control; b = reserpine control vs. test.

EPM is one of the most popular animal models of anxiety based on the study of spontaneous behavior. The anxiety-relieving effect has been evaluated using EPM tests. The behavioral parameters of EPM provide information about approach and avoidance behaviors and general activities in rodents. The most established parameters for assessing fear reflect avoidance of open weapons, such as number of times entering open weapons and total time spent at open weapons. The results are shown in Figure 2a,b. Analysis of the number of open weapons entered by the EPM test yielded the corresponding results. A significant statistical difference was found between the rutin I test (40 mg/kg) and the standard (*p* < 0.05). However, with respect to the dose of the rutin II test (80 mg/kg), no significant differences were found between the experimental group and the control group or the standard. The EPM study showed that rats treated with test rutin II (80 mg/kg) exhibited less fear of open arms and less fear of open arms over time. These behaviors were confirmed by the drug-induced behavior of the rutin rats as a measure of anxiety.

#### 2.2.2. Light-Dark Test

**Figure 3:**
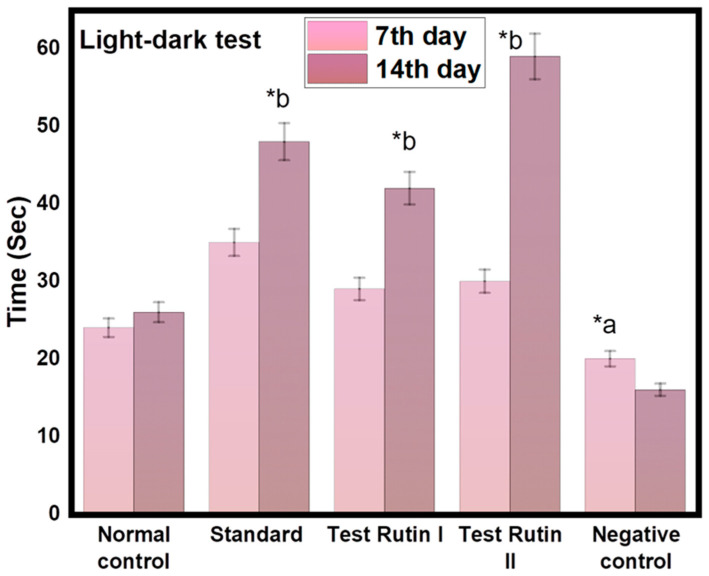
Effect of rutin pretreatment on antianxiety activity in rats using Light-dark test. The results are expressed as mean ± SD values (*n* = 6). The significance of the difference was determined by two-way ANOVA; * *p* < 0.05 vs. respective controls. a = normal control vs. reserpine control; b = reserpine control vs. test.

Light-dark tests (LD) are commonly used in rodents to detect unresolved anxiety behavior. They are based on a conflict between approach and avoidance between the desire to explore new areas and the aversion to bright and open spaces. Rutin II (80 mg/kg) significantly increased the time spent exploring bright spaces (*p* < 0.05, Figure 3). The results show that rats treated with rutin II (80 mg/kg) spent more time in the light and dark areas compared to the other groups. Other behavioral analyzes for anxiety revealed no significant differences between groups. The following graph shows a dose-dependent increase in time spent in light areas after administration of rutin (80 mg/kg), a potent anxiolytic. The increased time spent in bright areas indicates decreased anxiety.

#### 2.2.3. Open-Field Test

**Figure 4:**
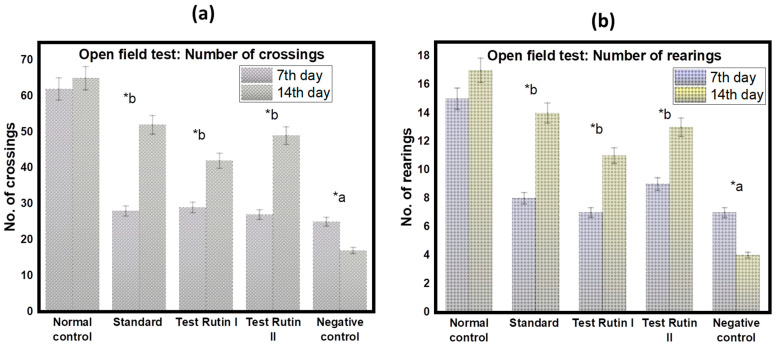
Antianxiety activity in rats after treatment using open field test; (**a**) Number of crossings; (**b**) Number of rearings. The results are expressed as mean ± SD values (*n* = 6). The significance of the difference was determined by two-way ANOVA; * *p* < 0.05 vs. respective controls. a = normal control vs. reserpine control; b = reserpine control vs. test.

Open-field tests serve as a general locomotion test and as a habit test. After treatment, a significant increase in the number of crossings (*p* < 0.05, Figure 4a) and number of rearing (*p* < 0.05, Figure 4b) were observed in rats treated with rutin (80 mg/kg) compared to control groups. Compared to the standard, the number of crossings and rearing were drastically reduced in the negative control rats.

### 2.3. Effect of Rutin Pretreatment on Antidepressant Activity

#### 2.3.1. Forced Swim Test

We evaluated for the first time whether rats were able to cope with stress and inescapable situations using both FST and TST. In these tests, the immobility time indicates the helplessness learned, indicating behaviors similar to those of depression [21]. We compared the FST behavior responses of rutin (40 mg/kg) and rutin (80 mg/kg) and examined the effects on helpless behavior. Interesting, after 14 days of treatment, the result of the rutin treatment group (80 mg/kg) was almost the same as that of the Standard and Normal. As a result, climbing accordingly (Test Rutin II: 98 s; Standard = 115 s; *p* < 0.05) and swimming (Test Rutin II: 142; Standard = 156 s; *p* < 0.05) increased. Increased immobility in FST is often used as a sign of behavioral despair. Increased FST shows that rats reduce depression at doses of 80 mg/kg. As a result, the test confirmed that rutin increased its dose, showing antidepressant effects.

#### 2.3.2. Tail Suspension Test (TST)

**Figure 5:**
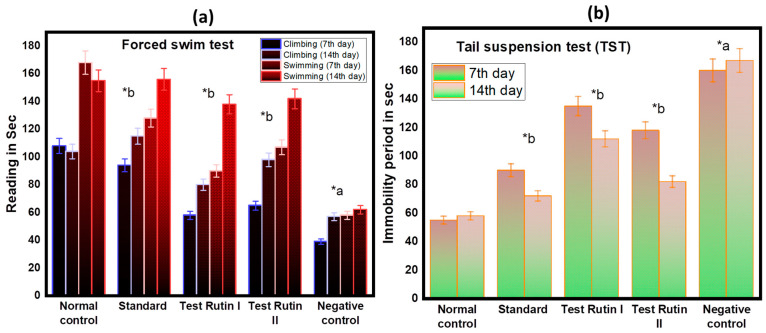
Effect of rutin pretreatment on antidepressant activity in rats (**a**) Forced swim test; (**b**) tail suspension test. The results are expressed as mean ± SD values (*n* = 6). The significance of the difference was determined by two-way ANOVA; * *p* < 0.05 vs. respective controls. a = normal control vs. reserpine control; b = reserpine control vs. test.

TST was also carried out to further confirm the lack of helplessness. In the TST, the mean immobility time for Rutin II (58 ± 12.24 s), Standard (72 ± 22.27 s) and Negative control (167 ± 24.57 s) after 14 days of treatment as shown in Figure 5b. Similar to FST, TST rats have a significantly longer immobility time than Wistar rats.

### 2.4. Effect of Rutin Pretreatment on Learning and Memory Activity

#### 2.4.1. Novel Object Recognition (NOR)

**Figure 6:**
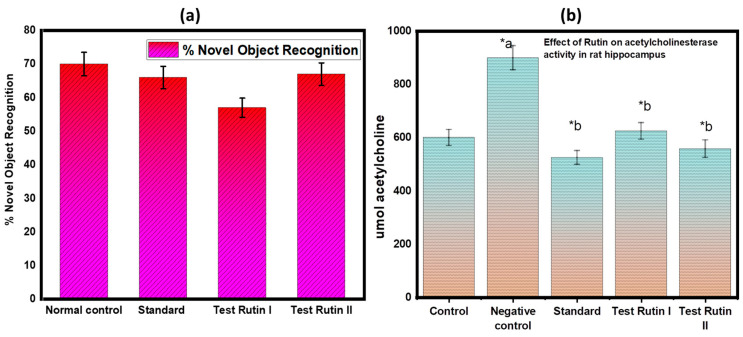
(**a**) Novel object recognition test; (**b**) In vitro assay for acetylcholinesterase activity. The results are expressed as mean ± SD values (*n* = 6). The significance of the difference was determined by two-way ANOVA; * *p* < 0.05 vs. respective controls. a = normal control vs. reserpine control; b= reserpine control vs. test.

Figure 6 a illustrates the results of the NOR assay for the nootropic action. In this study, after 14 days of therapy with various doses, the effect on memory function was evaluated. The recognition index (percentage) was calculated for the novel object and the findings showed statistically significant results with a *p* value of <0.05. Following the findings, pretreated rutin groups demonstrated a significant increase in the recognition index for a novel item compared to the control groups and standard treated groups.

#### 2.4.2. Effect of Rutin Pretreatment on In-Vitro Acetylcholinesterase Activity

AChE are very reliable, sensitive, and specific indicators of inflammation. Measurement of their activity may be valuable as a guide for forecasting the development of neurodegenerative disorders, as well as the prognosis and responsiveness to therapy in these conditions. The activity of AChE was measured in the hippocampus of rats (Figure 6b) in response to the given treatment.

### 2.5. Brain Histopathological Analysis

**Figure 7:**
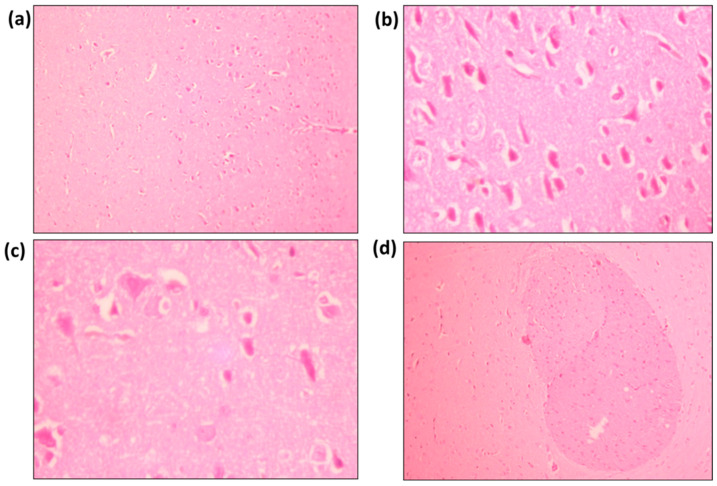
Histological features of (**a**) Standard; (**b**) Rutin I; (**c**) Rutin II; (**d**) Negative control.

Histological studies were performed in the forebrain region of the rats as shown in Figure 7. Standard brain parenchyma shows neurofibrillary tissue with purkinje cells, dendritic cells, and neuroglial cells. Very few congested vascular spaces and mononuclear inflammatory infiltrations are also seen within the neurofibrillary matrix are also seen Figure 7a. The brain parenchyma of rutin I show neurofibrillary tissue with purkinje cells, dendritic cells and neuroglial cells. In addition, congested vascular spaces and scattered mononuclear inflammatory infiltrations can be seen in Figure 7b. The brain parenchyma of rutin II shows neurofibrillary tissue with purkinje cells, dendritic cells and neuroglial cells. Congested vascular spaces and scattered mononuclear inflammatory infiltrations can also be seen in Figure 7c. The brain parenchyma of the negative control shows neurofibrillary tissue with well-demarcated dense eosinophilic areas. These eosinophilic areas consist of increased oligodendrocytes along with scattered purkinjee cells, dendritic cells and neuroglial cells. Large congested vascular spaces and sparse mononuclear inflammatory infiltrations within the neurofibrillary matrix Figure 7d.

## 3. Discussion

Drugs such as tricyclic antidepressants (TCAs), selective serotonin reuptake inhibitors (SSRIs), selective reversible inhibitors of monoamine oxidase A (RIMA), and specific serotonin-norepinephrine reuptake inhibitors (SNRIs) are commonly used in clinical pharmacological treatment [22]. Meanwhile, these drugs can cause cardiovascular toxicity, hypotension, sexual dysfunction, weight gain, and sleep disorders, among others [23]. These conditions provide an opportunity for alternative treatments for depression using medicinal plants and secondary metabolites. Previous studies have shown that flavonoids have a protective effect on the brain and can reduce depression-like behaviors [24,25,26].

Consequently, various pharmacological effects of rutin have been reported, including neuroprotective, antioxidant stress, anti-inflammatory, and nephroprotective effects. In 2017, Parshar et al. showed that rutin had antidepressant and anxiety-like effects in rats exposed to an unpredictable chronic stress paradigm [11]. In this study, we found that the administration of rutin to rats significantly reduced the time it took them to remain still in the FST.

The depression model in animals induced by Reserpine was used to carry out the study. Reserpine has been recognized as a psychotropic antihypertensive and anti-psychotic drug for more than 50 years. However, long-term use is associated with side effects caused by depression, such as depletion of monoamine brain concentrations. Therefore, the use of Reserpine for depression simulation in animals has been proven to be a correlation for studying depression in humans. Therefore, we chose an animal model for depressed and anxiety-like behavior caused by the depletion of monoamine by administration of Reserpine [27]. We have tried to verify the effectiveness of rutin’s antidepressants and anxiety relievers.

Behavioral tests such as OFT, TST, and FST are widely used to assess antidepressant and anxiety activity in animals. OFT measures the activity of searching in new environments and identifies behaviors similar to anxiety. At the same time, it is believed that FST and TST support depression behavior by stimulating mouse escape instincts and confirming whether active coping patterns are maintained. In these tests, increased activity and the reduction in motion indicate antidepressant and anxious effects of the drug. The study found that rats administered with Reserpine showed reduced central home time and initiation frequency in OFT and increased mobility in TST and FST. Therefore, our results indicate that rutin reduces the anxiety and despair caused by Reserpine, measured in OFT and FST/TST, respectively.

## 4. Materials and Methods

Chemicals: rutin (Sigma Aldrich, St. Louis, MO, USA), Reserpine, dimethyl sulfoxide, Tween-80 (were purchased from Sigma Aldrich, St. Louis, MO, USA. Amitriptyline (Dellwich Healthcare LLP, Ahmedabad, Gujarat, India), Ketamine (Themis Pharmaceuticals Ltd., Maharashtra, India), and xylazine (Med Vet, Mumbai, India) were purchased from commercial sources. The chemicals and solvents were of all analytical grade in pure quality. All chemicals were analytical grade and purchased from commercial sources.

### 4.1. Experimental Animals

The male/female Sprague-Dawley rats were weighing 180–240 g (each group contains 2 male and 4 female animals). The Standing Committee on Bioethic Research (SCBR-024-2022) Prince Sattam Bin Abdulaziz University, Al-Kharj, Ministry of Education, Kingdom of Saudi Arabia approved the studies. The animals were kept in a regulated cycle of 12 h of light and 12 h of darkness at a constant temperature (22 ± 2 °C) and relative humidity 55 ± 10%). The rats were accustomed to the cage for at least a week before starting the experiment. Water and food were provided ad libitum. Table 1 shows how doses were administered in this experiment. Reserpine was administered intraperitoneally to the rat at a dose of 0.5 mg/kg in PBS containing 0.1% dimethyl sulfoxide and 0.3% Tween-80) at a dose of 100 µL dose to induce anxiety and depression-like behaviors. Each drug was administered at a dose proportional to the animal’s body weight (mg of drug per kilogram of rat body weight).

#### 4.1.1. Experimental Procedures

- Changes in Body Weight and food Consumption in Reserpine-Injected Rats

Daily monitoring the weight and food consumption in rats. During the treatment period, food intake was determined by feeding rats a defined amount and measuring leftover food each morning (rats were individually caged). The weight and behavioral parameters of the rats were also monitored every day during treatment.

#### 4.1.2. Evaluation of Antianxiety Activity

- Elevated plus maze (EPM)

The EPM with two open arms (50 × 10 cm) and two closed arms (50 × 40 × 10 cm) and an open ceiling was 50 cm above the floor to study the behavior of antianxiety in animals. Throughout the experiment, rats were allowed to socialize. We took all possible measures to eliminate the possibility that the rice would be scared of anything other than the height of the plus maze. The dose administration program was adjusted so that each rat had to activate the EPM 60 min after vehicle and test material administration. Each rat was carefully placed in the center of the EPM with his head facing the open arm. Rat activity during the five-minute experiment was measured in two ways: (a) how often the rat went into the open arms and (b) how long it remained in the arms [28,29].

- Light-dark test

The light/dark device was a 30 × 30 × 30 cm wooden box with two chambers (dark chamber). The chambers were separated by a thin opaque wall. The rat could enter the dark chamber through a small hole in the wall. In the larger chamber, which was painted white, a light source was placed 27 cm above the floor. The smaller chamber was painted dark. Each rat was placed in the light chamber one hour after receiving the vehicle or test material, facing away from the door of the dark chamber. The latency for the first transition from the light to the dark chamber, as well as the time spent in the light zone, was measured during the 5-min test period [30,31].

- Open-field test

The open field equipment used consisted of 30 cm diameter plexiglass arenas with walls of 35 cm height. The arena consists of 32 squares; divided into the central (security area) of 8 squares, the peripheral (safety area) of 16 squares, and the middle zone of 8 squares. Each rat was placed in the center one hour after being administered the vehicle or test substance, and the following parameters were monitored for 5 min: (a) total number of crossings and (b) number of rearings [32,33].

#### 4.1.3. Evaluation of Antidepressant Activity

- Forced swim test (FST)

The test was performed according to the method of Porsolt et al. [34]. Animals are divided into different treatment groups and, as mentioned above, drugs are administered in different doses. After 30 min of treatment, each rat was placed in a plexiglass cylinder (25 cm height, 10 cm diameter containing water to a height of 10 cm at 25 °C) and observed for 6 min during rest (s). Rats were considered to be immovable when floating in stable water and make only small movements to prevent falling. The total duration of immobilization was recorded in the last 4 min of 6 min test [35].

- Tail suspension test (TST).

The test was carried out according to the methodology of Steru et al. [36]. A new group of animals was treated with various drugs, as shown in Table 1. In this case, 30 min after treatment, the rat was individually suspended in a retraction station placed 50 cm above the ground and placed with the help of an adhesive tape about 1 cm from the tail end. The total immobilization time was recorded during the last 4 min of the 6 min test. Animals are regarded as inanimate when they do not move their bodies or hang passively.

#### 4.1.4. Evaluation of Learning and Memory Activity

- Novel Object Recognition (NOR)

The size and processes of the NOR device are identical to those of Bhuvanendran et al. [37]. The behavioral test was performed between 9:00 and 18:00 during the red light. The object studied was a two-part transparent water bottle and a Lego toy similar to the bottle (new object). During the testing session, two different types of objects were displayed. They differ in texture, color, size, and shape. Assessment of these parameters consists of three stages: (i) usualization, (ii) training, and (iii) test. On the first day, each rat received about 10 min to learn about the open box without an object. On the second day, each rat was placed in an open field for five minutes and was free to examine two similar objects. After 60 min of training, the old tool was replaced with the new one and a two-minute test was performed on rats. The time spent on each object has been recorded. The open field box was cleaned between runs with 70% ethanol to minimize odor trace. The recognition index is calculated using the following formula: the ratio TB/(TA + TB). Where, [TA = time spent exploring familiar object A; TB = time spent exploring the novel object B] [32]. Exploring an object is defined as the smell or touch of an object with a nose or front foot. Turning or sitting on an object is not considered an exploration [37].

- In vitro assay for acetylcholinesterase activity (AChE)

The activity of AChE was carried out as described without any further modifications [38]. In this experiment, rats were sacrificed by decapitation and their brains were quickly removed and placed in reverse Petri plates. After dissection, the brain and hippocampus were weighed and divided equally into ten volumes of medium containing 10 mM Tris-HCl buffer, pH 7.2, and 160 mM sucrose. All homogeneous particles were centrifuged for 10 min and produced low-speed supernatants (S1). The hippocampus is homogeneous in 20 volumes in a medium and the homogeneous is centrifuged at 1000× *g*/15 min/4 °C. The percentage of S1 was used to determine the activity of AChE. The standard for measuring protein concentration in the sample is bovine serum albumin [39]. All samples were tested twice.

#### 4.1.5. Brain Histopathological Analysis

The animals treated with control and rutin were sacrificed after intravenous administration of ketamine at specific points of time. The brain was dismantled and fixed with the Bouin solution. After fixation, smaller fragments were processed using an automated tissue processor (Leica TP1020), dehydrated, and embedded in paraffin wax. Hematoxylin and eosin staining was performed on several 12-μm slices using a Leica Autostain-XL and a Microm HM360 automated microtome. Microscopic observation was performed under the LEICA DMLB and photographs were taken with the Leica DC 500 camera.

#### 4.1.6. Statistical Analysis

The data obtained are expressed in the format of average ± SEM and analysed in version 4.00 of the Graphic Pad Prism software. Data statistical analysis is carried out in significance of difference was determined by one-way ANOVA. *p*-values less than 0.05 (*p* < 0.05) were considered statistically significant.

## 5. Conclusions

Depression is the fourth leading cause of long-term impairment worldwide. It is one of the most common mental illnesses. Currently available data show that herbal medicines such as rutin may be useful in reducing symptoms of anxiety and depression. Our research has the potential to serve as a preclinical foundation that confirms the potential of rutin as an antidepressant that is capable of treating depression and anxiety-like behaviors that are produced by changes in monoamine levels. More research will be required to determine the mechanisms of action that govern antidepressant and anxiety-relieving effects.

## Figures and Tables

**Table 1:** Grouping of animals.

Groups	Subjects	Treatment Given
Group I	Normal control	Vehicle (water)
Group II	Standard	Reserpine (0.5 mg/kg ip) + amitriptyline (25 mg/kg, p.o.)
Group III	Test Rutin I	Reserpine (0.5 mg/kg ip) + Rutin (40 mg/kg p.o)
Group IV	Test Rutin II	Reserpine (0.5 mg/kg ip) + Rutin (80 mg/kg p.o)
Group V	Negative control	Reserpine (0.5 mg/kg ip)

## Data Availability

The data presented in this study are available on request from the corresponding author.

## Sample Availability

Samples of the compounds are not available from the authors.
